# Granular Disulfide-Crosslinked Hyaluronic Hydrogels: A Systematic Study of Reaction Conditions on Thiol Substitution and Injectability Parameters

**DOI:** 10.3390/polym15040966

**Published:** 2023-02-15

**Authors:** Luis Andrés Pérez, Rebeca Hernández, José María Alonso, Raúl Pérez-González, Virginia Sáez-Martínez

**Affiliations:** 1 Instituto de Ciencia y Tecnología de Polímeros (ICTP-CSIC), c/Juan de la Cierva, 3, 28006 Madrid, Spain; 2 i+Med S. Coop. Parque Tecnológico de Álava, Albert Einstein 15, Nave 15, 01510 Vitoria-Gasteiz, Spain

**Keywords:** injectable hydrogel, hyaluronic acid, thiol, microgels, granular hydrogels

## Abstract

Granular polymer hydrogels based on dynamic covalent bonds are attracting a great deal of interest for the design of injectable biomaterials. Such materials generally exhibit shear-thinning behavior and properties of self-healing/recovery after the extrusion that can be modulated through the interactions between gel microparticles. Herein, bulk macro-hydrogels based on thiolated-hyaluronic acid were produced by disulphide bond formation using oxygen as oxidant at physiological conditions and gelation kinetics were monitored. Three different thiol substitution degrees (SD%: 65%, 30% and 10%) were selected for hydrogel formation and fully characterized as to their stability in physiological medium and morphology. Then, extrusion fragmentation technique was applied to obtain hyaluronic acid microgels with dynamic disulphide bonds that were subsequently sterilized by autoclaving. The resulting granular hyaluronic hydrogels were able to form stable filaments when extruded through a syringe. Rheological characterization and cytotoxicity tests allowed to assess the potential of these materials as injectable biomaterials. The application of extrusion fragmentation for the formation of granular hyaluronic hydrogels and the understanding of the relation between the autoclaving processes and the resulting particle size and rheological properties should expand the development of injectable materials for biomedical applications.

## 1. Introduction

Hyaluronic acid (HA) is a naturally occurring anionic glycosaminoglycan that can be found in almost all body tissues and fluids, such as synovial fluid and eye vitreous humor. It is a linear non-sulfated polysaccharide composed of repeating units of the disaccharides D-glucuronic acid and N-acetyl-D-glucosamine. HA is part of the extracellular matrix and due to their high hydrophilicity (able to trap 1000 times the weight of water) provide structure to tissues and lubrication to the body [1]. The polymer can be rapidly degraded within the body by hyaluronidase and endothelial cells of the lymphatic vessels, being their biological half-life of 24 h [2]. In addition, HA presents different biological activity depending on the molecular weight. HA with molecular weight above 1000 kDa displays anti-apoptotic activity whereas HA fragments with mass below 200 kDa show strong pro-inflammatory properties [3]. In order to extent the half-life of HA, thus improving its stability into the human body, HA can be cross-linked through physical or chemical bonds to obtain HA hydrogels yet maintaining the biocompatibility and biodegradability that characterize the native material [4].

In a recent review, the formation of HA hydrogels through different chemical reactions carried out under physiological conditions was discussed as a means to obtain HA chemical hydrogels for biomedical applications. Some of the chemical reactions include click chemistry and the formation of dynamic (reversible) covalent bonds [5]. Within this context, chemical modification of HA with dynamic thiol moieties attracts a great interest due to the intrinsic properties of thiolated HA, such as mucoadhesiveness, swelling capacity, stability and biocompatibility [6]. In fact, thiol compounds can mimic endogenous polymers such as proteins with their cysteine subdominants. In addition, thiols can be used as anchor groups for bioactive compounds, or to perform crosslinking reactions for the preparation of scaffolds for tissue engineering through thiol-disulfide exchange reactions, thiol—epoxy reactions, thiol Michael additions and thiol-ene/yne “click” reaction [7]. Polymers modified with thiol moieties can also undergo in situ gelation trough inter- and intra-chain disulfide bond formation which, in addition, can be formed with cysteine-rich proteins such as mucins or keratins providing a firm adhesion to numerous biological surfaces [8,9]. However, disulfide bond formation by thiol-oxidation reaction at physiological conditions is characterized by a slow reaction due to the necessity of deprotonation of the thiol group (pKa of thiol of 8–10) to react with oxygen [10]. Nevertheless, the reaction kinetic can be accelerated through the employment of initiators or oxidizing agents to form the hydrogel which might compromise the biocompatibility properties of the resulting hydrogels [11,12]. Disulfide-crosslinked hyaluronic acid hydrogels have been proposed as bioactive hydrogels to be potentially employed as injectable hydrogels in tissue engineering and drug delivery applications including 3D extrusion bioprinting [13,14].

Recent studies have proposed the employment of the so-called granular hydrogels as injectable materials [15,16,17]. Granular hydrogels consist of a dense aggregation of micro-gel particles that can be injected through a needle showing shear-thinning and self-healing behavior due the particle non-covalent interactions which allows ease of injection and then stabilization upon injection [18]. In addition, granular hydrogels present microscale porosity between particles that can improve cell invasion or diffusion of bioactive molecules. Different techniques can be used to form granular hydrogels, such as microfluidic devices, batch emulsions or mechanical fragmentation [19,20].

Herein, we report on the design on granular disulfide-crosslinked HA hydrogels for applications as injectable biomaterials, taking into account not only the effect of the degree of substitution on the formation of granular hydrogels, their injectability and rheological properties but also the effect of autoclaving sterilization. Storage and sterilization are important factors to consider in the design of hydrogel materials to ensure patient safety and because sterilization processes can have unexpected effects on the biological and mechanical behavior of the hydrogels [21,22]. To fabricate HA hydrogels, modification of hyaluronic acid with thiol groups was first carried out to obtain hyaluronic acid with different degrees of substitution. Even though the modification reaction of hyaluronic acid with thiol groups has been extensively reported in literature [23,24,25,26,27], to the best of our knowledge, no systematic study on the experimental parameters that affect the modification degree of HA with thiol groups has been carried out. Herein, HA was modified with thiol groups using two different reactants: cystamine hydrochloride (Cys) and 3,3′-dithiobis(propiono hydrazide) (Hyd). The effect of reaction parameters such as pH, time and ratio of reactants on the HA degree of substitution was fully elucidated and discussed as a function of the reaction mechanism. Then, hydrogels were formed using self-crosslinking of thiolated hyaluronic acid in air at 37 °C. Selected HA formulations were subjected to mechanical fragmentation through extrusion to form HA microgels due to its simplicity, speed and low cost and subjected to sterilization prior to injectability and rheological tests. Finally, the cytotoxicity of the resulting injectable granular hydrogels was ascertained.

## 2. Materials and Methods

### 2.1. Materials

Hyaluronic acid (HA) with molecular weight 0.5–0.75 MDa was purchased from Contipro. Cystamine dihydrochloride (Cys, Sigma Aldrich, St. Louis, MO, USA), 3,3’-dithiobis(propanoic dihydrazide) (Hyd, FisherScientific, USA) and dithiothreitol (DTT, Fluorochem, Hadfield, United Kingdom) were employed for HA modification with thiol groups. N-(3-Dimethylaminopropyl)-N′-ethylcarbodiimide hydrochloride (EDC, Fluorochem, Had- field, United Kingdom) and 1-Hydroxybenzotriazole hydrate (HOBt, Sigma Aldrich, St. Louis, MO, USA) were used as coupling reagents. Spectra/Por membranes (MWCO: 10 kDa) were employed for purification by dialysis. Phosphate-buffered saline (PBS, Sigma Aldrich P4417, St. Louis, MO, USA) was used as solution medium to form the hydrogels. 5,5′-Dithiobis (2-nitrobenzoic acid) (DTNB Ellman’s reagent, Sigma Aldrich, St. Louis, MO, USA), Ethylenediaminetetraacetic acid (EDTA, Sigma Aldrich, St. Louis, MO, USA) and Cysteina-HCl (Thermo fisher Scientific, USA) were used for the determination of thiol groups.

### 2.2. Hyaluronic Acid (HA) Modification with Thiol Groups (HA-SH)

Hyaluronic acid was modified with thiol groups using two different reactants, Cystamine hydrochloride and 3,3′-dithiobis (propiono hydrazide), in a two-step reaction. The modification was carried out according to an adapted experimental protocol reported in literature [28]. In brief, a solution of HA in phosphate-buffered saline (PBS) was prepared (1% *w/v*), then the molecule to be incorporated into the polymer chain (cystamine dihydrochloride, Cys or 3,3′-dithiobis (propiono hydrazide), Hyd) was added in excess molar with respect to HA carboxyl groups along with 1-Hydroxybenzotriazole hydrate (HOBt), which was added in an excess molar ratio with respect to HA carboxyl groups. In order to evaluate the effect of pH on the HA substitution degree, the pH of the solution was adjusted from 4.1 to 7.5 through the addition of NaOH (1M) and HCl (1M). Afterwards, EDC was added to the solution in molar excess with respect to HA carboxyl groups to initiate the coupling reaction step. The reaction was allowed to proceed overnight at room temperature. Then, the mixture was dialyzed against deionized water for several days. To proceed with the second reaction step, the pH of the mixture was adjusted to 8.5. Finally, dithiothreitol (DTT) was added to the solution in excess against initial HA carboxyl groups to reduce the disulfide bond. The reaction was left to run overnight. The pH of the solution was adjusted to 3.5 and then it was dialyzed against acidified deionized water for several days. The final products were freeze-dried for storage.

The effect of the molar ratio COOH/EDC on the degree of substitution of hyaluronic acid modified with Cys was evaluated with a slight modification of the synthesis protocol described above. In brief, a solution of HA (1% *w/v*) in phosphate-buffered saline (PBS) was prepared, reactants Cys and HOBt were added to the solution at selected excess molar ratios and the pH of the solution was adjusted to 4.1 using HCl (1M). Then, EDC was added to initiate the coupling reaction. Three samples were prepared with COOH/EDC molar ratios, 1/1, 1/2.5 and 1/3. After 2.5 h, the pH of the solution was adjusted at pH 9 and allowed to proceed overnight at room temperature. The mixture was dialyzed against deionized water for several days. To proceed with the second reaction step, the pH of the mixture was adjusted to 8.5 and DTT was added to the solution to reduce the disulfide bond. The reaction was left to run overnight. Finally, the pH of the solution was adjusted to 3.5 and then it was dialyzed against acidified deionized water containing NaCl 0.1 M for several days. The final products were freeze-dried for storage.

Reaction yields (%) were calculated by dividing the amount of final product (grams) by the initial amount (grams). The substitution degree % (%SD) of the resulting HA samples were determined through proton nuclear magnetic resonance spectroscopy (1H-NMR) in a Varian System-500 equipment. Samples were dissolved in deuterated water and spectra were taken at 25 °C.

### 2.3. Preparation of Disulfide Crosslinked HA Hydrogels

To form bulk hydrogels, thiolated HA material was first dissolved in acidified PBS to a final concentration of 15 mg/mL (1.5% *w/v*). Then, the pH of the solution was adjusted to 7.4 and homogenized using an Ultra-Turrax (3 min, 14,000 rpm). Gel formation after 24 h was evaluated by the inverted vial test.

The same protocol was used to prepare samples to monitor the reaction progress. After homogenization with Ultra-Turrax, solutions were poured onto 20 mm diameter Teflon molds and allowed to crosslink in air at 37 °C. Samples were prepared in triplicate for each composition. The initial weight of the sample (amount of solution) was fixed at 0.950 g per sample. The initial weight of the sample allowed us to determine the shrinkage of the sample during the crosslinking reaction (Equation (1)):

(1) $$Shrinkage\;\%=\frac{\left(m_{i}-m_{x}\right)}{m_{i}}\cdot 100$$

where m_i_ is the initial mass of the precursor solution before gelation and m_x_ is the mass after completion of the reaction.

In addition, to monitor gel formation, the amount of unreacted thiol groups was determined at specific time intervals through the Ellman method. A UV-Vis spectrometer PerkinElmer Lambda 35 was employed to measure the characteristic absorption peak at 412 nm. The reaction yield was calculated from Equation (2) [29].

(2) $$Reaction\;Yield\;\%=\frac{n_{0}-n_{t}}{n_{0}}\cdot 100$$

where n_0_ is the initial concentration of thiol groups in the solution and n_t_ is the concentration of unreacted thiol groups at a certain time.

Disulfide crosslinked HA hydrogels were denoted as HA-S-S-XX, where XX refers to the degree of substitution of the initial thiolated HA material.

### 2.4. Characterization of Disulfide Crosslinked HA Hydrogels

The stability of the resulting HA hydrogels was evaluated against simulated physiological conditions (PBS buffer, pH 7.4 at 37 °C) by measuring the mass change of the sample at specific intervals of time. Before weighing, samples were removed from the medium and the excess of buffer solution on the hydrogel surface was absorbed gently with paper. Then, the sample was immersed in fresh PBS buffer. All experiments were performed in triplicate. The mass change of the hydrogel was calculated through Equation (3):

(3) $$Mass\;change\;\%=100\%-\left(\frac{W_{0}-W_{t}}{W_{0}}\cdot 100\right)$$

W_t_ is the mass weight at specific intervals of time and W_0_ is the initial weight for hydrogels after complete gel formation. 

The morphologies of the hydrogels were characterized by scanning electron microscopy (SEM) in a Hitachi S-8000 instrument (Tokyo, Japan) operating in transmission mode at 1 kV accelerating voltage. Two different drying techniques were employed to prepare the samples for SEM experiments: freeze-drying and supercritical CO_2_ drying. For freeze-dried samples, hydrogels were frozen in liquid nitrogen prior to lyophilization. For supercritical CO_2_ drying, hydrogels were immersed in water/ethanol dilutions starting from water/ethanol mixtures (90/10%*v*/*v*) to progressively reaching a 100% ethanol solution [30]. After phase-change, samples were dried in a Thar R100W supercritical CO_2_ reactor at a temperature of 35 °C and a pressure of 100 bars during 90 min.

### 2.5. Fabrication and Characterization of Sterilized Granular HA Hydrogels

For the preparation of disulfide crosslinked hyaluronic acid granular hydrogels, bulk samples were granulated by extraction fragmentation. A hydrogel was loaded into a syringe and then, sequentially extruded by hand through needles of decreasing diameters (18G, 20G and 22G) following a procedure reported elsewhere [31]. The granular hydrogels were loaded within 2 mL closed syringes, centrifuged to eliminate entrapped air bubbles and sterilized. Sterilization process was performed by autoclaving using a Systec V-65 autoclave (Linden, Germany). The sterilization step was performed for 15 min at 121 °C. Finally, the samples were stored at 4 °C. 

Particle size diameter was determined for swollen particles in PBS suspensions at room temperature using optical profilometry (Zeta Instrument profilometer model Z-20; KLA Company, California, USA). Toluidine Blue O was used as colorant and ImageJ software was employed to determine particle size and distribution. 

The extrusion force required to evaluate injectability of the hydrogels was determined through assays carried out in a Dynamometer PCE/FB200 coupled to a motorized vertical test bench SAUTER TVM 5000N230N at a compression speed of 40 mm/min. The syringes were coupled to a 21G × 40 mm needle to carry out the tests. 

Rheological analysis of extruded hydrogel samples was performed using an advanced rotational rheometer from TA instruments, model AR-G2 (DE, US) with 20 mm Cross-Hatched Stainless Steel Peltier Plate geometry. All experiments were carried out at 25 °C. Storage (G’) and loss (G”) modulus were measured by an oscillatory frequency sweep with frequency 0.01–100 Hz and 1% strain. Critical strain was assessed by oscillatory strain sweep, carried out at a frequency of 1.0 Hz and a strain ranging from 0.01 to 250%. Critical strain was determined as onset analysis in which a line is fit to the plateau region and the drop-off region.

To study the shear thinning behavior of the granulated hydrogel, a flow sweep test from 0.01 to 100 s^−1^ were performed. To evaluate the recovery behavior, shear recovery experiments were performed applying low (1%) and high (250%) strains periodically at a frequency of 1.0 Hz.

Parameters such as osmolality, refractive index, pH and in vitro cytotoxicity were evaluated into the sterilized samples. Osmolality of the sterilized hydrogels was determined using the measurement of freezing point depression by the cryoscopic osmometer Gonotec Osmomat 3000D (Berlin, Germany). Distilled water and solutions of 300 and 850 mOsmol·kg^−1^ were used as blank and calibration solutions, respectively. Refractive index of granulated hydrogels was recorded by a refractometer Optika 2WAJ refractometer at room temperature. The sample was extruded through the needle and deposited over the refractometer. Then, in order to form a continuous layer, some drops of the buffer PBS were deposited into the extruded hydrogel. The pH values of the samples were measured at room temperature using a 781 Metrohm pH-meter (Switzerland).

### 2.6. Biological Tests

Cytotoxicity of sterilized hydrogels was studied by the extraction method on cultures of Adult Human Dermal Fibroblasts (HDF-a, Science Cell 2320) primary cells according to ISO 10993-5:2009, Biological evaluation of medical devices—Part 5: Tests for in vitro cytotoxicity. Briefly, HA-S-S-HA hydrogels and controls were prepared according to UNE-EN-ISO 10993-12:2003. Hydrogels were incubated in culture media for 24 h, at 37 °C under constant stirring to obtain the initial extracts (100%), which were further diluted to 75%, 50% and 25%.

HDF primary cells were seeded at a density of 20.000 cells/well in 96-well plates and cultured for 24 h at 37 °C and 5% CO_2_. Next, media was replaced by new media (positive control) or the different extracts (samples). Wells with culture media were run alongside and were used as blanks. After 24 h, cell viability was measured using MTT procedure. Absorbance at 570 nm was measured using a microplate reader (Varioskan Flash, Thermo Fisher Scientific). After blank correction, cell viability was calculated according to Equation (4).

(4) $$Cell\;viability\;\left(\%\right)=\frac{A_{570nm\;X}}{A_{570nm\;PC}}\cdot 100$$

where A_570nm X_ is the absorbance read of the different extracts, while A_570nmPC_ is the average value of the positive control reads. Three independent experiments were performed with six replicates per experiment. Mean values ± standard deviations are reported. One-way analysis of variance (ANOVA) and Tukey’s post hoc test were used to determine statistically significant differences (* *p* < 0.05;) between the means.

## 3. Results and Discussion

### 3.1. Hyaluronic Acid (HA) Modification with Thiol Groups (HA-SH)

**Scheme 1:**
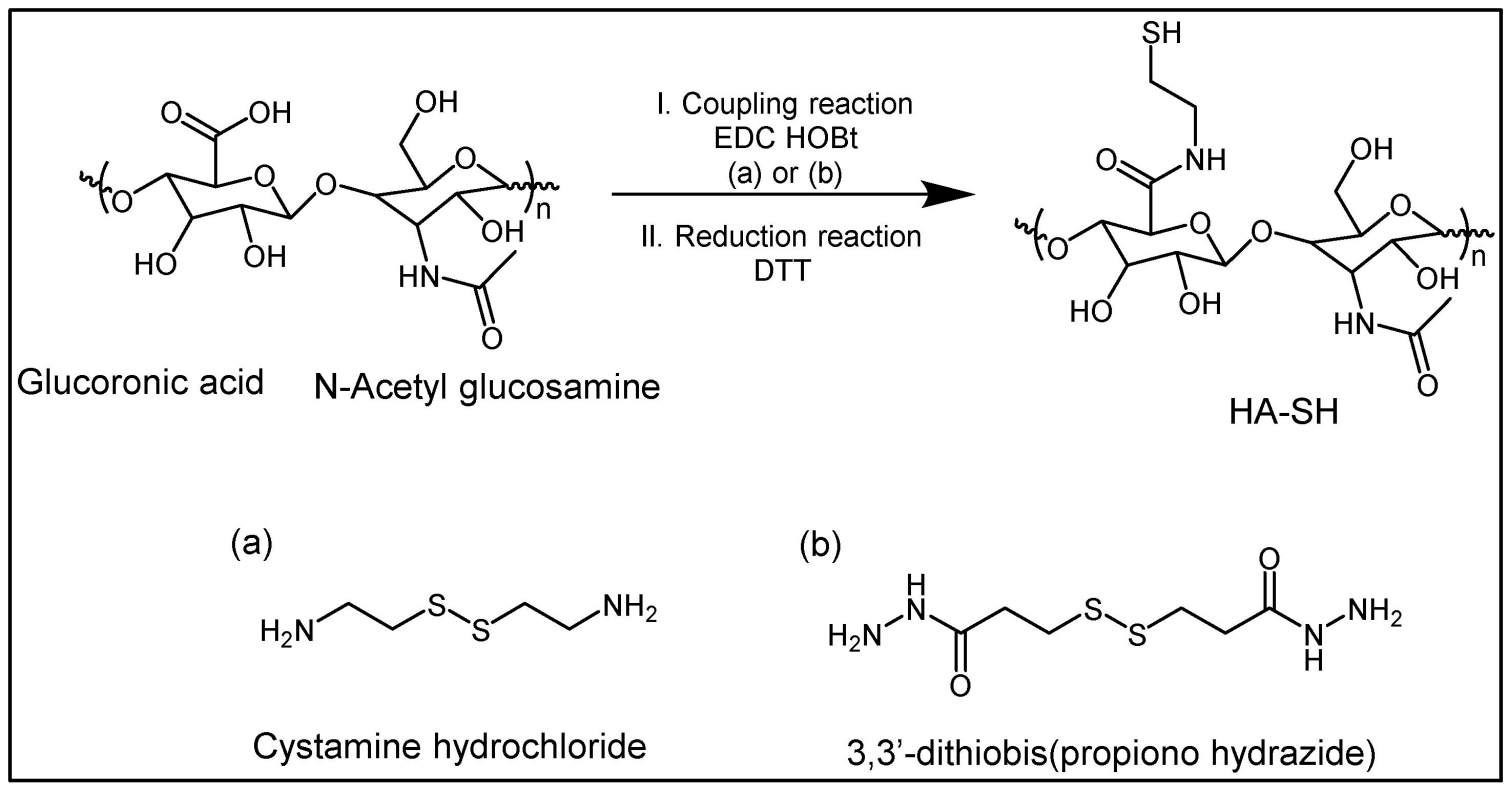
Hyaluronic acid modification, reaction scheme using two different molecules: (**a**) cystamine dihydrochloride (Cys) and (**b**) 3,3′-dithiobis(propiono hydrazide) (Hyd).

The modification of HA was carried out by two-step reaction, as shown in Scheme 1. First, an amidation coupling reaction was carried out to functionalize hyaluronic carboxyl groups with amines or hydrazides groups. EDC and HOBt were used as carboxyl activator and as a coupling reagent, respectively. In a second step reaction, the disulfide bond was reduced with DTT to yield thiol groups.

Reaction yields defined as the amount of the modified material in relation to the initial one was determined. Yields of 85–90% were obtained for all samples.

**Figure 1:**
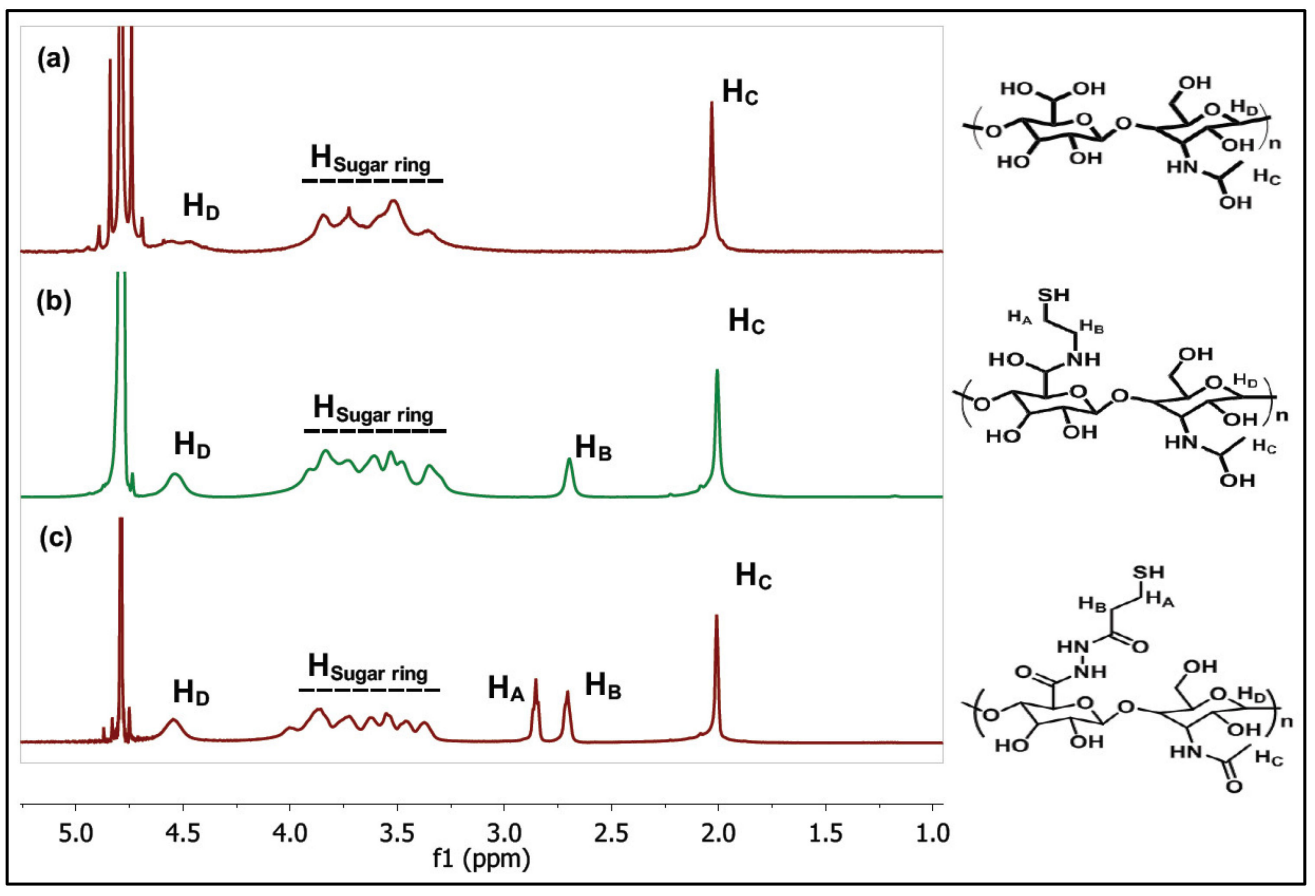
^1^H-NMR spectrum of (**a**) non-modified HA, thiolated HA modified with (**b**) cystamine dihydrochloride and Cys and (**c**) 3,3′-dithiobis (propiono hydrazide), Hyd.

The substitution degree % (%SD) of the thiol group into the polymer chain was evaluated by ^1^H-NMR. Figure 1 shows representative ^1^H-NMR spectra of thiolated hyaluronic acid, obtained from Cys (Figure 1b) and Hyd (Figure 1c) and non-modified hyaluronic acid (Figure 1a). The ^1^H-NMR spectrum corresponding to non-modified HA shows a peak located at 2.0 ppm, δH_C_, that can be assigned to the protons of the methyl group in the HA N-acetyl group. The signals corresponding to the polysaccharidic rings are found within the region 3.2–4 ppm. In ^1^H-NMR spectrums of modified HA, the appearance of peaks located at 2.7 ppm (δH_A_) corresponding to thiolated HA modified with Cys and 2.8 (δH_B_) and 2.7 ppm (δH_A_) in the ^1^H-NMR spectrum corresponding to HA modified with Hyd demonstrated the substitution of HA with thiolated side chains.

The substitution degree was calculated through Equation (5):

(5) Substitution degree % %SD=IδHB2IδHC3·100

where I_δHB_ corresponds to the integration area of protons located at 2.7 ppm and I_δHC_ are the integration area of protons located at 2.0 ppm. 

**Figure 2:**
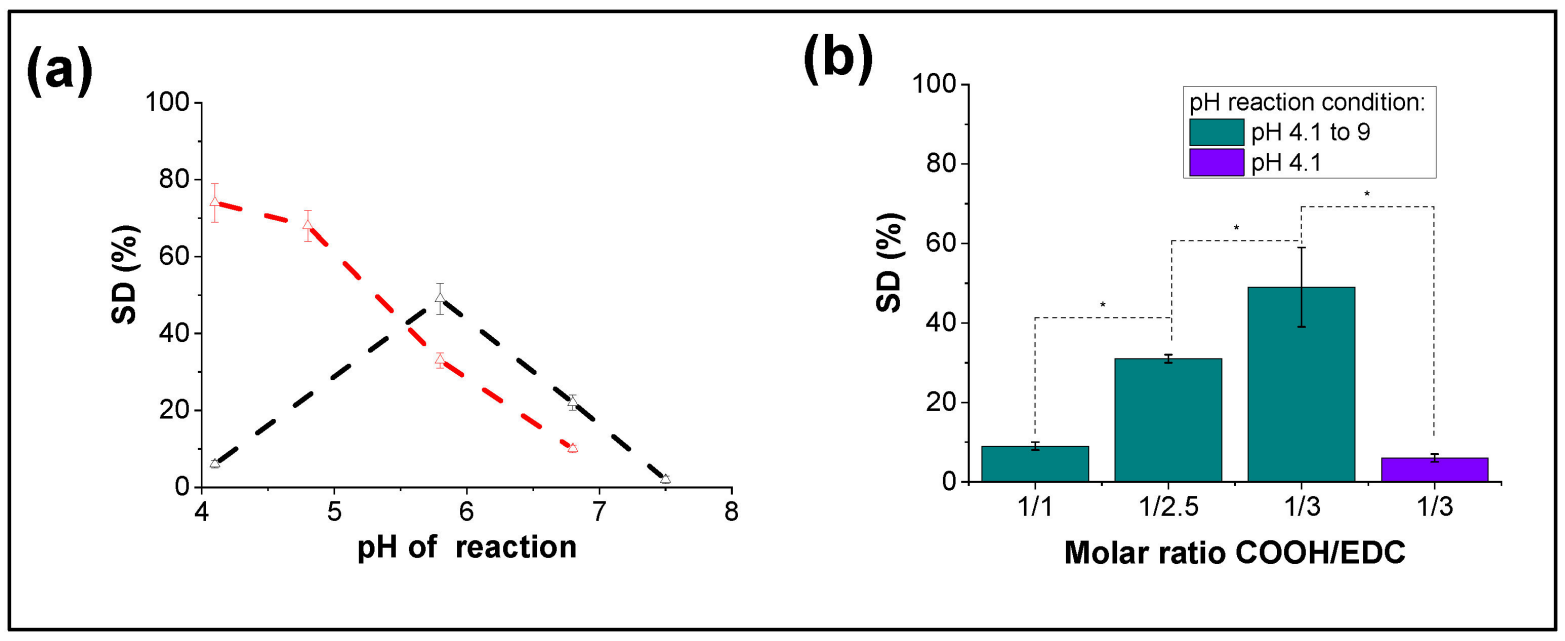
Substitution degrees (SD) of HA samples modified with thiol groups (**a**) as a function of the reaction pH using two different reactants, cystamine hydrochloride (3/1 molar ratio (EDC/COOH), black triangles) and 3,3′-dithiobis(propiono hydrazide) (2/1 molar ratio (EDC/COOH), red triangles), dashed lines are a guide for the eye and (**b**) as a function of the molar ratio for samples modified with cystamine hydrochloride. The purple bar represents the value obtained employing a constant pH 4.1 throughout the reaction of modification.

The HA substitution degree as a function of the pH of the coupling reaction was studied for HA reacted with cystamine hydrochloride and 3,3′-dithiobis(propiono hydrazide), the results are shown in Figure 2a.

For thiolated-HA samples modified with cystamine, the substitution degree first increases to values ~50% at pH 5.8 and then decreases to values ~5% at pH 4.1. The increase in %SD in the pH range 7.5 to 5.8 can be attributed to an increase in the reactivity of EDC reagent which shows a more stable form at neutral pH conditions [32]. As the pH further decreases (from 5.8 to 4.1), amide formation (i.e., anchoring of the thiol group) is not favored due to the protonation of the amine group into the cystamine reagent (pKa values of ≈ 10–11 [33]), which reduces the group reactivity thus preventing the nucleophilic attack [34]. When the reaction was carried out with 3,3′-dithiobis(propiono hydrazide), the degree of modification increased as the pH becomes more acidic due to the fact that hydrazide groups maintain their nucleophilicity at pH 4–7 because of their lower pKa values (usually 2–4) [35].

To further optimize the first reaction step and increase the HA substitution degree, the molar ratio COOH/EDC was varied, and reaction pH was increased to 9 after 2 h from the reaction onset in order to increase the reactivity of the amine group of the cystamine molecule once the intermediate HA-HOBt was formed. As can be observed in Figure 2b, the substitution degree increases from 9 ± 1% to 49 ± 10% with an increase in the amount of EDC from a 1/1 COOH/EDC ratio to a 1/3 COOH/EDC ratio. In addition, the change of pH from 4.1 to 9 during the reaction greatly improves the obtained SD%. In fact, HA samples modified at a constant pH of 4.1 at a 1/3 COOH/EDC ratio showed SD% values of only 6 ± 1%. It is concluded that both the molar ratio EDC/COOH and the pH of the medium are significant factors that control the HA substitution degree in agreement with previous results found in the literature [36].

Thiolated HA samples reacted with 3,3′-dithiobis (propiono hydrazide) with substitution degrees of 65%, 30% and 10% were used for the study of hydrogel formation, effect of sterilization process and cytotoxicity assays and the results are described in the following sections.

### 3.2. Monitoring of the Reaction of HA Disulfide Crosslinking as a Function of HA Degree of Substitution

As previously reported, thiol-modified HA can be crosslinked at physiological conditions through oxidation of the thiol groups into disulfide bonds as previously reported [37]. For our study, we intend to monitor disulfide crosslink formation at room temperature for thiolated-HA with three different SD%, HA-S-S-65%, HA-S-S-30% and HA-S-S-10% over long times to establish a relation between the substitution degree of thiolated HA, the kinetics of the crosslinking reaction and the hydrolytic stability over time of fully crosslinked disulfide HA hydrogels. The three samples under study formed gels after 24 h in inverted vial tests.

**Figure 3:**
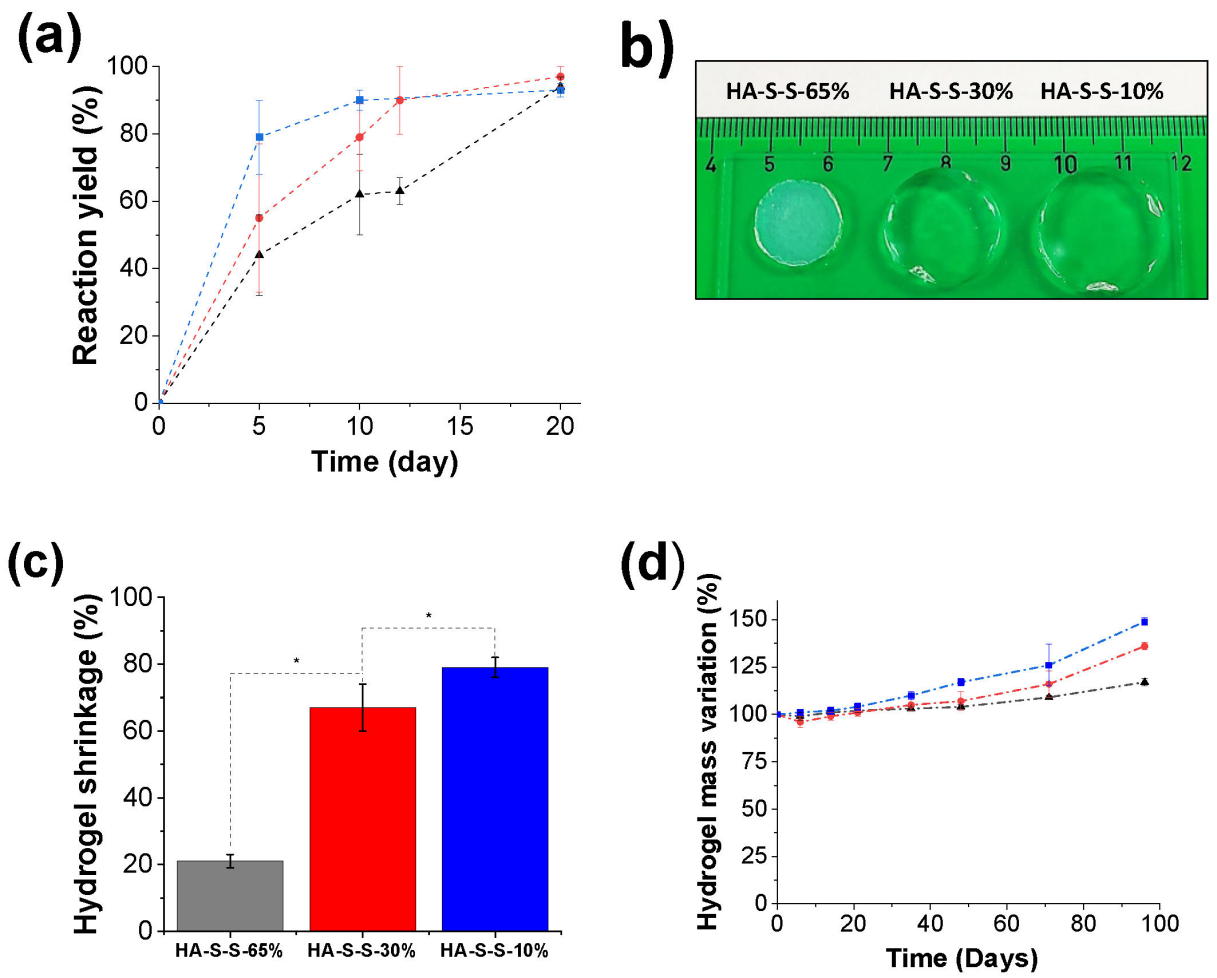
(**a**) Monitoring of hydrogel formation as a function of time (**b**) Representative image of disulfide crosslinked hydrogels and (**c**) Shrinkage of samples after complete reaction; (**d**) Hydrolytic stability of hydrogels, PBS pH 7.4, 37 °C. Samples represented: HA-S-S-65% (triangle, black color), HA-S-S-30% (circle, red color) and sample HA-S-S10% (square, blue color).

Samples prepared in Teflon molds were used to monitor the reaction through the consumption of thiol groups determined by Ellman’s method, Equation (2). The results are shown in Figure 3a. For the three samples under study, disulfide formation is very slow, and samples need several days to reach plateau reaction yields~ 90–95%. As the SD% increases, the disulfide formation rate is slower. HA-S-S-65% reached reaction yields of ~94% after 20 days whereas samples HA-S-S-30% and HA-S-S-10% reached reaction yields of ~90% after 12 and 10 days, respectively. This could be attributed to the fact that a higher number of thiols might hinder the mobility of the polymer chains as the polymer network is formed, hence, the rigidification of the network might hinder the crosslinking reaction rate [38].

In addition, all samples shrank during the S-S bond formation reaction. This effect was more evident for the sample HA-S-S-65% which showed an opaque appearance and a smaller diameter with respect to samples HA-S-S-30% and HA-S-S-10 (Figure 3b). The weight of the samples was controlled during the gel formation; constant weight values of 200 ± 30 mg, 640 ± 80 mg and 750 ± 30 mg were measured for sample HA-S-S-65% at day 20, HA-S-S-30% at day 12 and HA-S-S-10% at day 10, respectively, and then it reached a constant value. Shrinkage of the samples was determined by difference in weight after completion of the reaction (Equation (1)). The initial weight of samples was set to 950 mg; shrinkage of 79%, 33% and 21% were calculated for HA-S-S-65%, HA-S-S-30% and HA-S-S-10%, respectively (Figure 3c). The final concentration of the hydrogels was re-calculated considering the shrinkage of the sample and its weight, obtaining values of 73 ± 11 mg/mL, 23 ± 3 mg/mL and 19 ± 1 mg/mL for samples HA-S-S-65%, HA-S-S-30% and HA-S-S-10%, respectively. This result can be directly correlated with the degree of substitution of hyaluronic acid, that is, samples with a higher number of thiol groups will result in hydrogels with more crosslinking points and hence, higher shrinking.

After complete disulfide crosslinking reaction, the hydrolytic stability of the hydrogels over time was evaluated by mass change in buffer PBS, pH 7.4 at 37 °C (Figure 3d). All samples under study showed an increase in weight over time (100 days studied). The increase in weight could be attributed to an increase in water content into the sample due the partial degradation of cross-link points (greater freedom of the network) and the highly hydrophilicity of the HA causing a dissolution of the hydrogel [39]. In addition, the water uptake presented a correlation with the degree of modification of the hyaluronic acid, samples with a lower degree of modification/disulfide bond such as HA-S-S-10% showed a higher increase in weight than samples HA-S-S-30% and HA-S-S-65%, obtaining an increase of 149 ± 4%, 136 ± 2% and 117 ± 2%, respectively.

Finally, the morphology of the hydrogels was studied using scanning electron microscopy (SEM). Two methodologies were employed to obtain dry hydrogels, i.e., a solid whose dispersed phase is air: freeze-drying (Cryogel) and supercritical CO_2_-drying (Aerogel). Freeze-drying technique tends to produce samples with a macro-porous structure, characterized by large and irregular pores due to the water crystal produced when it freezes, whereas CO_2_ supercritical drying tends to produce samples with a nano-porous structure. This technique avoids the collapse of the material thus preserving the network structure [40].

**Figure 4:**
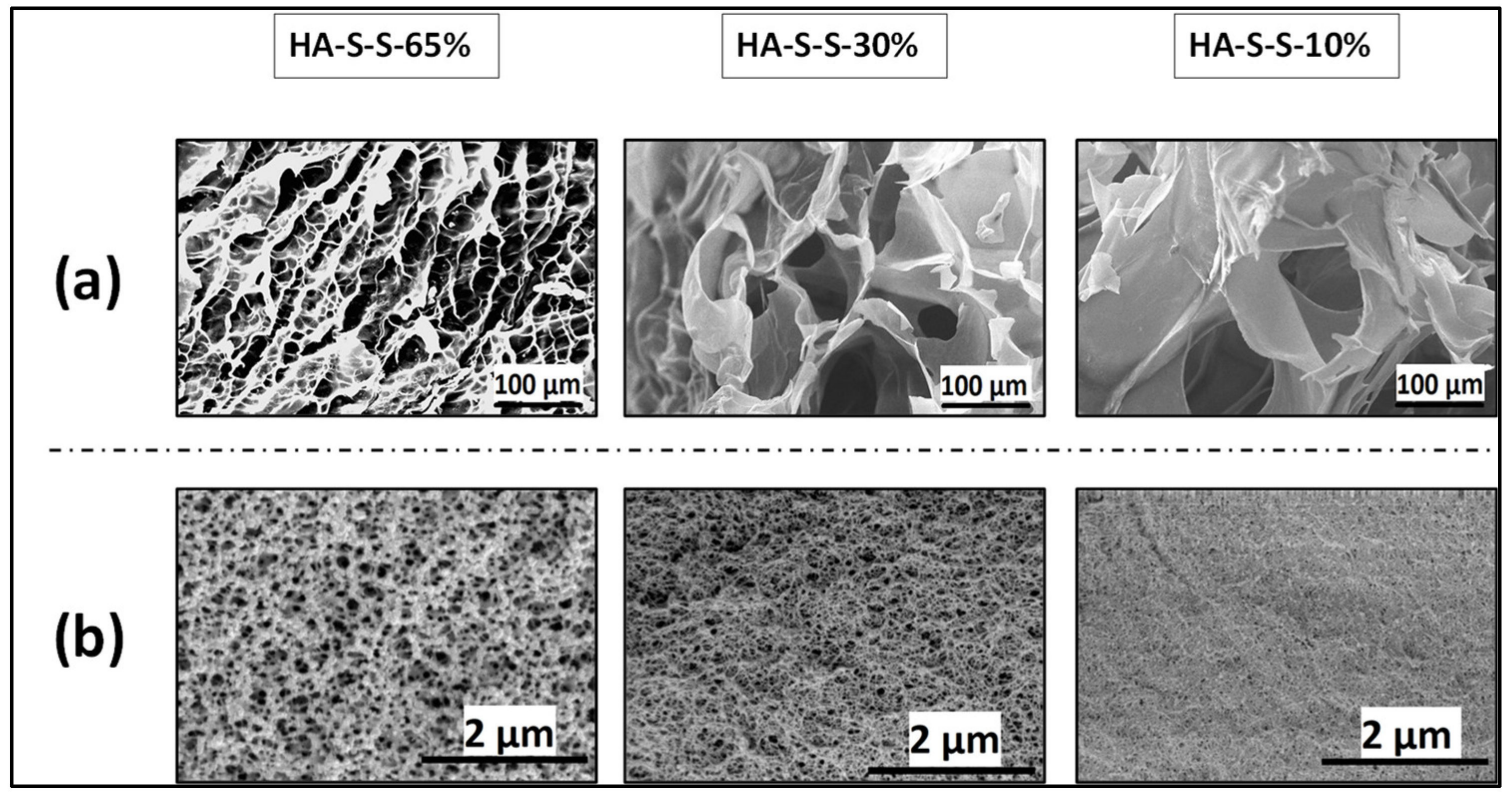
Morphology characterization of dry hydrogels: (**a**) thiolated HA cryogels, obtained from freeze-drying (×250 magnification); (**b**) thiolated HA aerogels, obtained from CO_2_ supercritical drying (×25.0k magnification).

As observed in Figure 4a, cryogels with highly interconnected porosity have been obtained for all the samples under study with pore size in the micro range of 20 ± 6 µm, 84 ± 20 µm and 165 ± 48 µm for HA-S-S-65%, HA-S-S-30% and HA-S-S-10%, respectively. It can be seen an inverse trend between solid component and porosity, cryogels derived from hydrogels with higher crosslinking degree (HA-S-S-65%) has a smaller pore size and greater wall thickness than the sample with less crosslinking degree (HA-S-S-10%). This trend could be related first to the variation in the polymer concentration upon crosslinking. That is, samples with a higher crosslinking ratio present a higher shrinkage and hence an increase in polymer concentration. Such an increase in the polymer concentration might also hinder water crystallization which might result in the lower pore size observed after freeze-drying [41,42]. In contrast, images corresponding to hyaluronic acid aerogels obtained through CO_2_ supercritical drying show a nanoporous morphology which is much more evident for the samples HA-S-S-65% and HA-S-S-30%, as can be observed in Figure 4b. For sample HA-S-S-10%, the morphology observed could be due to a higher volume shrinkage of the sample occurring during the ethanol-water phase change, originated from a lower crosslinking and polymer concentration. In fact, previous attempts in the literature to obtain HA aerogels without crosslinkers using CO_2_ supercritical drying technique and ethanol and acetone as solvents showed that aerogel formation was only possible at low pH (1.5 and 2.5). No aerogels were formed at pH 6 as the HA is deprotonated and no interactions between chains can be produced (the network cannot be formed), so the polymer solution cannot withstand the water-ethanol phase change [43,44].

### 3.3. Disulfide Crosslinked HA Granular Hydrogels Fabrication and Size Characterization

**Figure 5:**
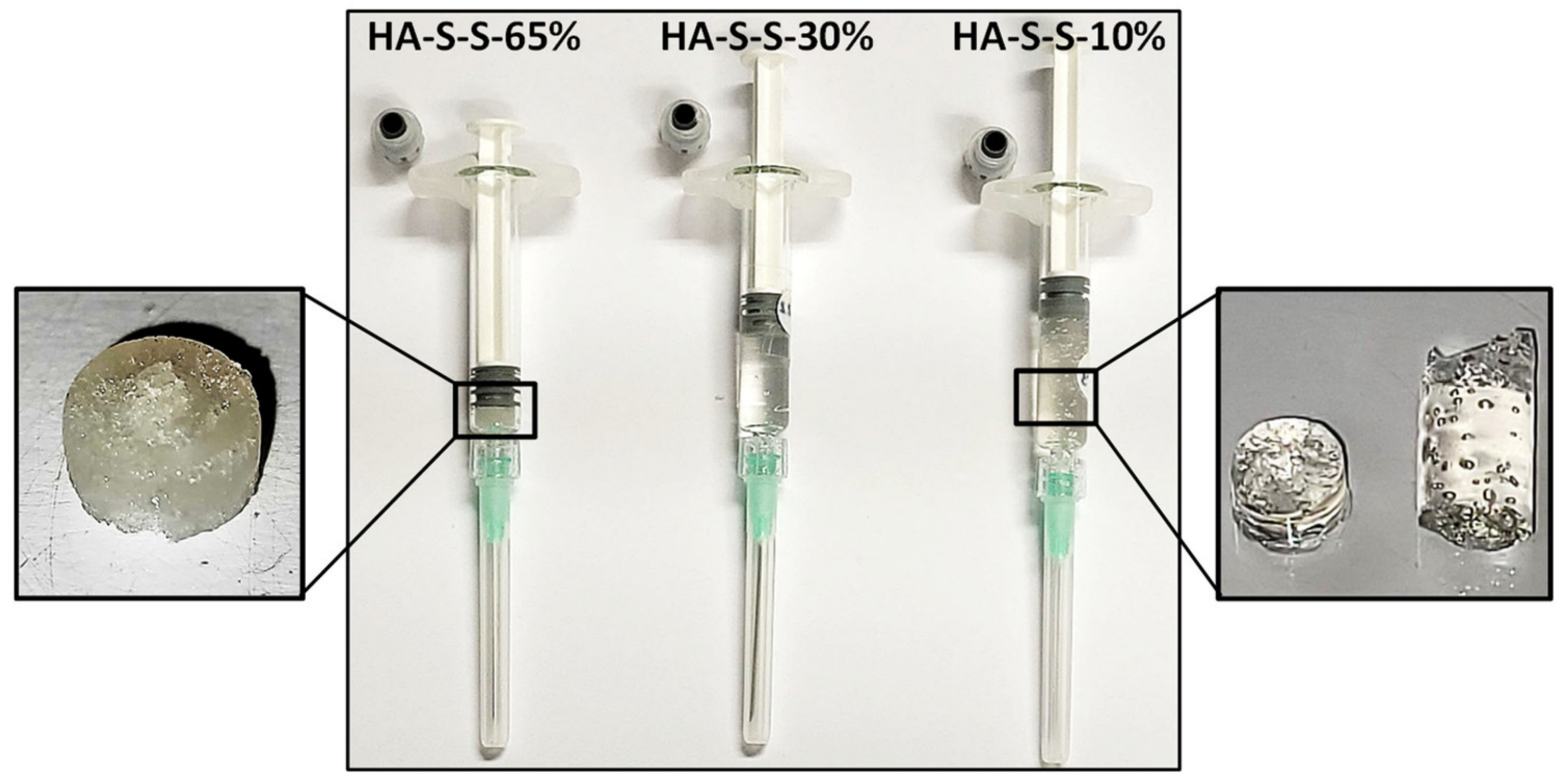
Central image: syringes loaded with hydrogels after autoclaving sterilization process: HA-S-S-65%, HA-S-S-30% and HA-S-S-10%. Side images: visual appearance of the hydrogels outside the syringe after sterilization.

In this section, hyaluronic acid “microgels” were formed by extrusion fragmentation method from “bulk” polymers hydrogels obtained from samples HA-S-S-65%, HA-S-S-30% and HA-S-S-10% with crosslinking degrees of ~95%, and then sterilized by autoclaving process in order to determine the final properties under real processing conditions. It is important to note that after the granulation/sterilization steps, a certain degree of compaction/recomposing of the hyaluronic acid “microgels” was observed within the syringes employed for sterilization (Figure 5). Such an effect was less pronounced for the sample HA-S-S-65% where some granulation could still be observed after sterilization. The effect of compaction/recomposing could be attributed to the reaction of residual unreacted thiol groups into the sample or a thiol exchange between disulfide bonds as a result of temperature and pressure.

**Figure 6:**
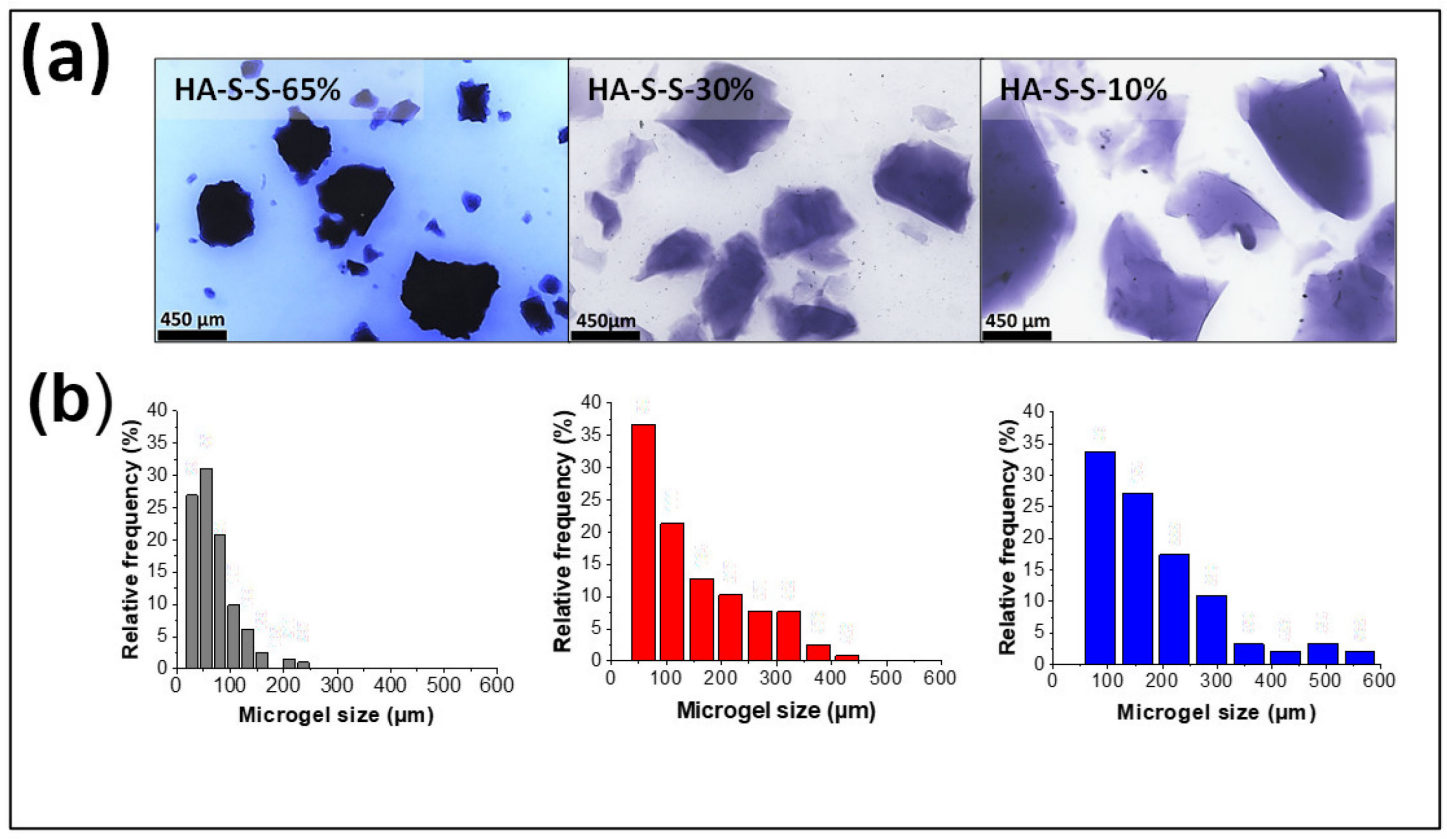
(**a**) Representative images of microgel particles in suspension, buffer PBS, pH 7.4 at room temperature; (**b**) Particle size distribution. (From left to right: HA-S-S-65%, HA-S-S-30% andHA-S-S10%).

After sterilization, samples were injected through a 21G × 40 mm needle and the size and distribution of the particles in a PBS suspension were evaluated. Extrusion fabrication technique leads to the formation of irregularly shaped microgels with particle size values of 71 ± 42 µm, 147 ± 98 µm and 189 ± 121 µm for HA-S-S-65%, HA-S-S-30% and HA-S-S-10%, respectively (Figure 6a). A general trend between the particle size and crosslinking degree can be observed, that is, the extrusion technique leads to smaller particle sizes as the degree of crosslinking of the pristine hydrogel increases. This effect may be due to the fact that less crosslinked materials could reach higher deformations without breakage when passing through the needle and therefore they fragment less. For all the samples under study, a heterogeneous size distribution could be observed (Figure 6b).

### 3.4. Rheological Properties and Injectability Studies of Granular Hydrogels

**Figure 7:**
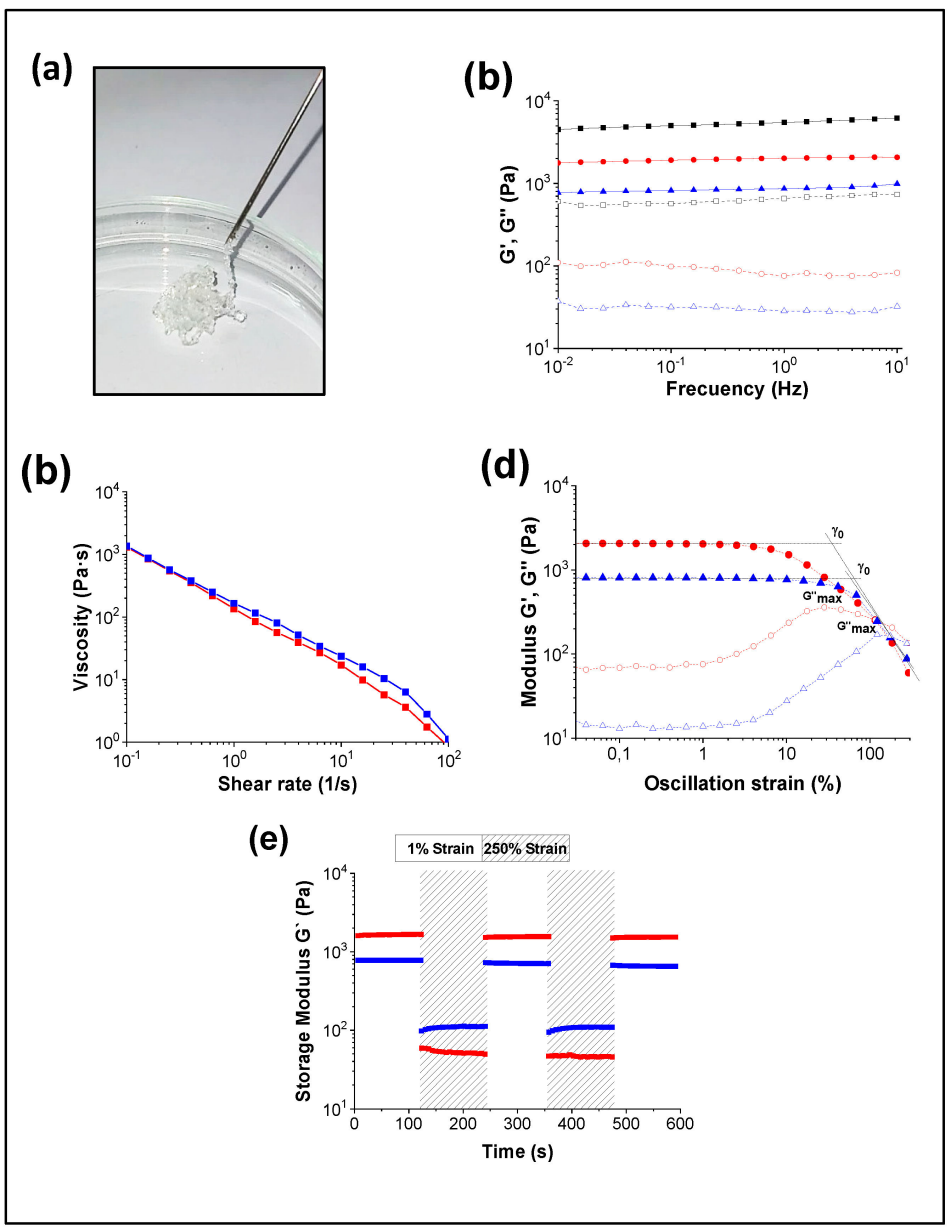
(**a**) Representative image of a granular HA-S-S-10% hydrogel being extruded; (**b**) Representative curves of frequency sweeps, storage moduli (G’) and loss moduli (G’) as a function of frequency, 1% of strain; (**c**) Representative flow curves, viscosity vs. shear rate sweep; (**d**) Representative strain sweeps, storage moduli (G’) and loss moduli (G’) as function as function of oscillatory strain, 1 Hz and (**e**) Representative dynamic strain tests alternating 0.1% and 250% strain at short time. Samples represented: HA-S-S-65% (triangle, blue color), HA-S-S-30% (circle, red color) and sample HA-S-S10% (square, blue color).

Hydrogels intended as injectable biomaterials require hydrogel flow through relatively narrow outlets determined by the syringe and the needle diameters. For granular hydrogels, their response to flow is important as individual microgel diameters can be within the range of the size scale of needle diameters. For the samples under study herein, injectability and rheological properties were assessed after autoclaving sterilization. Samples HA-S-S-10% and HA-S-S-30% were extrudable through a 21G needle resulting in filaments with structural integrity, as observed in Figure 7a, which shows the visual appearance of an extruded filament corresponding to sample HA-S-S-10%. Sample HA-S-S-65% did not form a homogeneous filament when extruded through the syringe. In addition, at first, only water could be extruded through the syringe thus further increasing the concentration of the sample HA-S-S-65% and thus preventing the determination of the extrusion force. Samples HA-S-30% and HA-S-S-10% required extrusion forces of 39 ± 6 and 88 ± 14 N, respectively, for their injection. Average maximum manual force was reported to be 79.8 N (males: 95.4 N, females: 64.1 N [45]); however, both extrusion forces deviate from the clinical standards values consider as a maximum for manual injection of 30 N [46,47].

The study of the rheological properties of granular hydrogels formed through fragmented microgels is of paramount importance for the development of their applications as injectable biomaterials and 3D extrusion bioprinting [20]. In fact, previous results in the literature have shown that granular hydrogels produced through extrusion processes present a significantly higher mechanical moduli when compared to those formed with spherical microgels. This has been attributed to the irregular shape of the microgel particles that promotes enhanced interparticle contact and interdigitation among the particles [31]. For the samples under study herein, oscillation frequency sweep tests showed a gel-like behavior for all samples. This gel state is characterized by a storage modulus (G′) higher than the loss modulus (G′′) and that both modulus are independent of frequency in the range under study [48]. The results show that the storage modulus of these granular hydrogels is greatly dependent on the crosslinking degree of the pristine hydrogel, increasing from G′ = 800 ± 60 Pa for the sample HA-S-S-10% to 2000 ± 300 Pa and 5200 ± 1000 Pa for the samples HA-S-S-30 and %HA-S-S-65%. The high elastic modulus observed for the sample HA-S-S-65% can also be attributed to its higher shrinkage and concentration of the sample, as previously reported [31,49].

Injectability properties of samples HA-S-S-30% and HA-S-S-10% were further explored through flow tests (Figure 7c). Both samples showed a typical shear-thinning behavior characterized by a decrease in viscosity with the shear rate. In addition, no significant differences were found when comparing the overall viscosity of both formulations having similar behaviors against the flow of the material. This may be related to similar irregular particle geometry thus resulting in a similar hydrogel packing and flow resistance. 

Next, self-healing properties were investigated. Oscillatory strain sweeps (Figure 7d) showed that both samples display a linear viscoelastic regime characterized by the independence of G′ and G″ on the strain and yield up to a critical strain, γ_0_, above which samples show a viscoelastic liquid-like behavior followed by apparent shear-thinning. Sample HA-S-S-30% and HA-S-S-10% exhibit critical strain values of ~23% and ~50%; therefore, for our study the sample that is less crosslinked (HA-S-S-10%) can withstand higher oscillation strains before gel disruption with respect to the sample HA-S-S-30%. Interestingly, a peak in the loss modulus G″ was observed for the samples during yield strain as shown in Figure 7d, G″_max_ values of 360 Pa at 30% of strain, and 170 Pa at 120% of strain was observed for samples HA-S-S-30% and HA-S-S-10%, respectively. These results point to a higher ability of the sample HA-S-S-30% to dissipate energy as a result of a higher number of disulfide bonds. This behavior, that has been reported in literature for other granular hydrogels, is known as weak-strain hardening [18,50]. It is directly related to the energy dissipation due to the breakage of a complex structure and related to gel rearrangements occurring in the transition to a fluid-like state [51,52].

To further investigate the self-healing behavior of both hydrogels, several step-strain tests were carried out in order to assess gel recovery, first at a low strain (γ = 1%) and then at a high strain (γ = 250%) (Figure 7e). Both samples showed complete recovery in the storage moduli after the application of 2 cycles of low and high oscillatory deformation.

### 3.5. Biocompatibility of Sterilized Granular Hydrogels

The concentration of the hydrogels was re-calculated considering the shrinkage of the sample. The values were 73 ± 11 mg/mL, 23 ± 3 mg/mL and 19 ± 1 mg/mL for samples HA-S-S-65%, HA-S-S-30% and HA-S-S-10%, respectively. Parameter such as pH and osmolality have gained attention into injectable hydrogels since they can affect cell viability and proliferation by apoptosis [53]. Physiological accepted pH values are found between 7.0–7.5, while optimal osmolality should be between 285–480 mOsmol·kg^−1^ range, from plasma osmolality (European Pharmacopeia value) to that of synovial fluid [54,55]. In addition, UNE-EN-ISO 15798:2013 sets a tolerable osmolality range of 200–400 mOsmol·kg^−1^ along with a pH range of 6.8–7.6 for ophthalmic implants [56]. 

To evaluate any harmful effect on the cells, the pH and osmolality of the hydrogels were evaluated after the sterilization process. The hydrogels presented pH values of ~7.4 for all samples. Regarding osmolality, values of 296 ± 10, 374 ± 4 and 353 ± 12 mOsmol·kg^−1^ were obtained for HA-S-S-65% HA-S-S-30% and HA-S-S-10%, respectively. All compositions studied present acceptable value ranges of pH and osmolality, as previously referred. Refractive indexes of the granular hydrogels were examined in order to study their applicability in ophthalmological applications. Sample HA-S-S-30% and HA-S-S-10% showed refractive index of 1.34 ± 0.2% and 1.33 ± 0.2%, respectively. Hydrogel HA-S-S-65% could not be measured due to the opacity of the sample. These values are almost identical to that in human vitreous, 1.33 [57].

**Figure 8:**
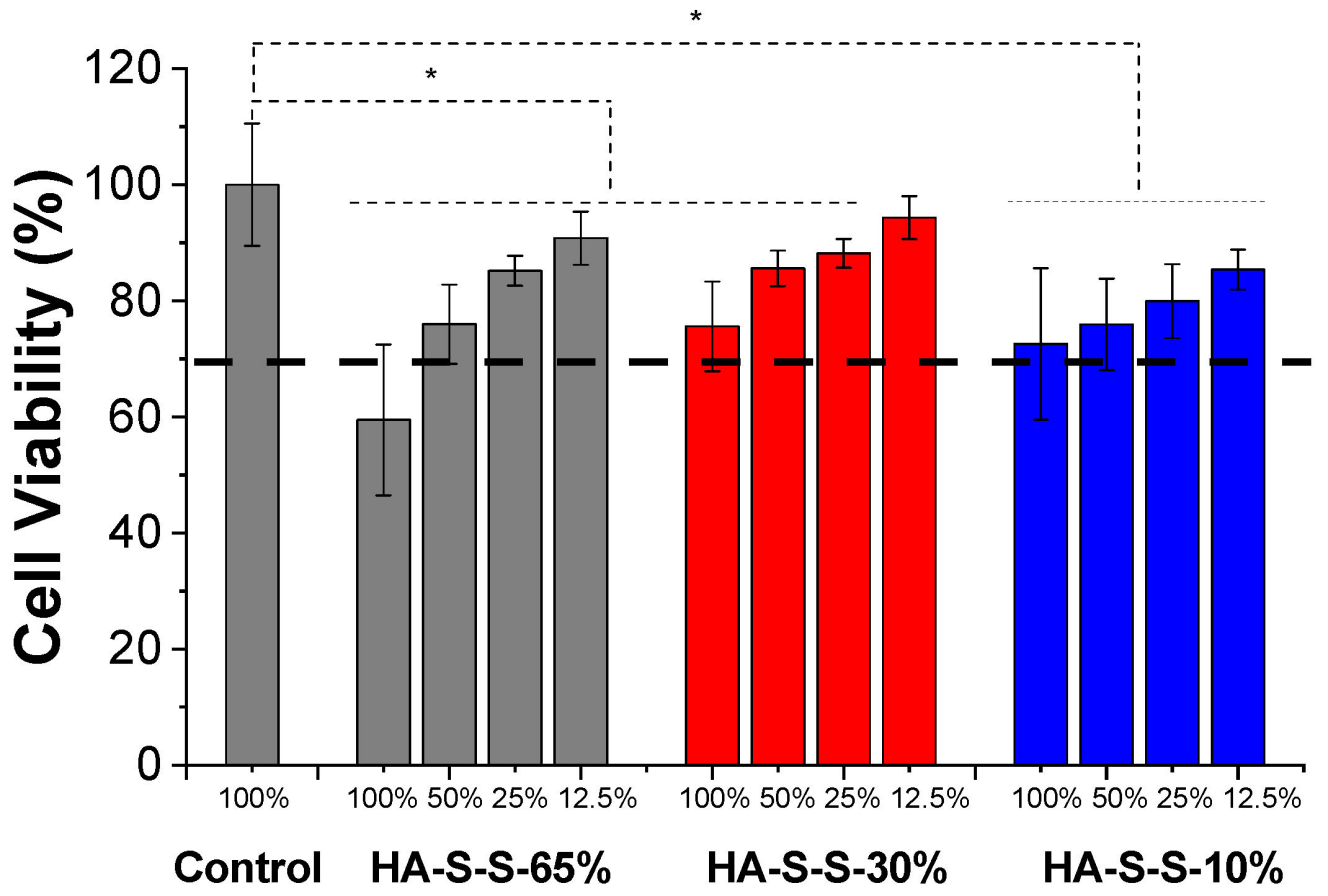
MTT cytocompatibility. Cytotoxic profiles of hydrogels after 24 h of incubation. ANOVA statistical significance (* *p* < 0.05).

In vitro *cytotoxicity test* was carried out based on UNE-EN-ISO 10993-5:2009, Figure 8. The standard states that a reduction of more than 30% in the cell is considered to have cytotoxic effect [58]. Cell viability of 59.5 ± 13.0, 75.6 ± 7.7 and 72.6 ± 13.1 were obtained at 100% extract for samples HA-S-S-65%, HA-S-S-30% and HA-S-S-10%, respectively (Figure 8). All extracted tested for the three samples showed a decrease (*p* < 0.05) in cell viability compared with the positive control sample. Samples HA-S-S-30% and HA-S-S-10% displayed non-cytotoxic behavior showing cell viabilities above 70%.

### 3.6. Conclusions

Herein, we investigated for the first time, the suitability of disulfide crosslinked hyaluronic acid granular hydrogels fabricated through extrusion fragmentation to be employed as injectable materials. As a first step, a systematic study about the influence of pH and reagents ratio on the degree of substitution with thiol groups was carried out with cystamine hydrochloride and 3,3’-dithiobis(propanoic dihydrazide). The results showed that at low pHs, the substitution reaction of HA with hydrazide groups gave rise to thiolated HA with higher SD% (74 ± 5%) compared to thiolated HA obtained through reaction with amine groups (6 ± 1%). Hence, samples reacted with hydrazide were chosen for further characterization.

Gel formation kinetics and shrinkage of thiolated HA samples were found to be highly dependent on the SD%. The higher the SD%, the longer the time to reach crosslinking yields above 90% and the higher the shrinkage of the resulting gels which, in turn, resulted in hydrogels with smaller pore sizes and higher hydrolytic stability. It is important to note that the formation of a nanoporous morphology was observed for disulfide crosslinked hyaluronic hydrogels subjected to supercritical drying for which a higher SD% (samples HA-S-S-65% and HA-S-S-30%) allowed to retain the porous structure after ethanol-water phase change.

Extrusion fragmentation gave rise to the formation of HA microgel particles whose size decreased with the increase of SD%, being 71 ± 42 µm, 147 ± 98 µm and 189 ± 121 µm for HA-S-S-65%, HA-S-S-30% and HA-S-S-10%, respectively. Autoclaving resulted in the compaction/jamming of the HA particles back into a macroscopic macrogel as a consequence of the presence of dynamic disulfide bonds. Gel microparticles with a higher crosslinking degree show less compaction after sterilization; thus, reducing the required extrusion force for injectability being 39 ± 6 N for sample HA-S-S-10% and 88 ± 14 N for sample HA-S-S-30%. Finally, disulfide crosslinked hydrogels obtained from HA with SD% of 30 and 10% were found to be non-cytotoxic making these formulations attractive candidates for further preclinical testing. Further experiments currently in progress will seek to optimize injection forces needed to inject granular disulfide hydrogels through combination of hydrogels obtained from HA with different modification degrees.

## Data Availability

The data presented in this study are available on request from the corresponding author. The data are not publicly available due to confidentiality issues.

## Abbreviations

Cys, cystamine hydrochloride; DTNB, 5,5′-Dithiobis (2-nitrobenzoic acid); DTT, dithiothreitol; EDC, N-(3-Dimethylaminopropyl)-N′-ethylcarbodiimide hydrochloride; EDTA, ethylenediaminetetraacetic acid; G′, storage modulus; G′′, loss modulus; HA, hyaluronic acid; HDF, Human Dermal Fibroblasts; HOBt, 1-Hydroxybenzotriazole hydrate; 1H-NMR, nuclear magnetic resonance spectroscopy; Hyd, 3,3′-dithiobis(propiono hydrazide); PBS, Phosphate buffered saline; PE, Polyethylene; SD, substitution degrees; SEM, Scanning Electron Microscopy.
